# Time estimation and beta segregation: An EEG study and graph theoretical approach

**DOI:** 10.1371/journal.pone.0195380

**Published:** 2018-04-06

**Authors:** Amir Hossein Ghaderi, Shadi Moradkhani, Arvin Haghighatfard, Fatemeh Akrami, Zahra Khayyer, Fuat Balcı

**Affiliations:** 1 Cognitive Neuroscience Lab., Department of Psychology, University of Tabriz, Tabriz, Iran; 2 Iranian Neuro-wave Lab., Isfahan, Iran; 3 Department of Physics, Amirkabir University of Technology, Tehran, Iran; 4 Department of biology, North Tehran Branch, Islamic Azad University, Tehran, Iran; 5 Department of Genetic, Tehran medical sciences branch, Islamic Azad University, Tehran, Iran; 6 Faculty of Health Management and Information, Iran University of Medical Science, Tehran, Iran; 7 Department of Psychology, University of Isfahan, Isfahan, Iran; 8 Department of Psychology, Koç University, Istanbul, Turkey; Duke University, UNITED STATES

## Abstract

Elucidation of the neural correlates of time perception constitutes an important research topic in cognitive neuroscience. The focus to date has been on durations in the millisecond to seconds range, but here we used electroencephalography (EEG) to examine brain functional connectivity during much longer durations (i.e., 15 min). For this purpose, we conducted an initial exploratory experiment followed by a confirmatory experiment. Our results showed that those participants who overestimated time exhibited lower activity of beta (18–30 Hz) at several electrode sites. Furthermore, graph theoretical analysis indicated significant differences in the beta range (15–30 Hz) between those that overestimated and underestimated time. Participants who underestimated time showed higher clustering coefficient compared to those that overestimated time. We discuss our results in terms of two aspects. FFT results, as a linear approach, are discussed within localized/dedicated models (i.e., scalar timing model). Second, non-localized properties of psychological interval timing (as emphasized by intrinsic models) are addressed and discussed based on results derived from graph theory. Results suggested that although beta amplitude in central regions (related to activity of BG-thalamocortical pathway as a dedicated module) is important in relation to timing mechanisms, the properties of functional activity of brain networks; such as the segregation of beta network, are also crucial for time perception. These results may suggest subjective time may be created by vector units instead of scalar ticks.

## Introduction

Time in classical physics is scaled by an electron transition frequency. The frequency of the microwave spectral line emitted by cesium atoms is used as a reference in a cesium standard or cesium atomic clock. According to this scale, time is a scalar variable with an accumulative property. A series of psychophysiological studies has been also considered subjective time with scalar properties [1–4]. This idea, typically referred to as the Scalar Expectancy Theory (STE), suggests that there is a linear relation between psychological magnitude of perceived time and objective clock reading of time [5]. In agreement with STE, Weber’s law states that the variability of time estimations has a linear relation with duration of intervals [1]. The information-processing variant of STE [2] is a prominent example of the pacemaker-accumulator based models of interval timing. Models that fall in the pacemaker-accumulator family assume that a modular internal clock is involved in time estimation and perception [4,6–9]). In this framework, the internal clock ticks as a classical cesium clock and the differences between the time that runs with internal clock and physical clock has been raised by differences in parameters such as: the number of pulses in pacemaker, storage of accumulator, and decision making processes.

On the flip side, modern physics suggests that time is a vector with direction or arrow [10] and time can be added to other similar vectors such as the space unit vectors [11]. As a matter of fact, the fundamental inconformity between classical and modern conceptualization of time is consequent of considering time as a vector. More precisely the classical conceptualization of time does not presume directionality and additivity of time to space vectors, and treats time as a scalar generally independent from speed, movement and information (entropy) whereas time in the modern conceptualization is strongly related to speed and information. Although alternative time perception models have been presented, asserting that subjective time can be accounted for without considering a dedicated internal clock, the vector property of physical time has been typically ignored. These models assume alternative functional architectures such as neural state transitions involved in time perception [12–14]. According to some of these accounts temporal information could just be an epiphenomenon which can be considered as a byproduct of other fundamental events such as energy spent during neural processing in the brain [12].

However, the present conceptualizations are unable to account for certain empirical facts about time perception. For instance, the properties that are treated as hallmarks of interval timing (e.g., scalar property) are violated in the case of some individuals [15] (but see [16] for dependencies of results on time scale and tasks utilized). An important differentiation that is often made in order to resolve some of these discrepancies is treating the timing system not as a unitary function, but rather as multiple functions (e.g., sub-second timing vs. supra-second timing vs. circadian timing; [17]).

Many human activities in natural settings and modern life last for several minutes (e.g., eating, listening to music, cooking). Long durations, typically referred to as cognitive time (a part of a “supra-second system”), are associated with general aspects of cognition such as consciousness, attention, and working-memory processes [16]. Consequently, cognitive time is strongly amenable to the effect of various contextual factors [18] and internal states such as personality, emotional state, arousal, cognitive load, and mood [19,20].

However, psychological and physiological studies in the area of interval timing have focused on the judgment of durations in the seconds to minutes range and they rarely address time intervals that last several minutes. This is partly due to the practical issues (e.g., number of trials that can be achieved in a test) and partly because minutes long intervals are implicitly assumed to rely on complex integration of multi-modal functions.

Most of electrophysiological studies were also performed to investigate short durations using event related potentials (ERPs) approach and by the frequency analysis [21]. In the frequency domain that is intended in this study, delta (1–4 Hz) and beta (13–30 Hz) EEG/MEG oscillations have been recently mentioned as EEG oscillatory rhythms that reflect neural processing of time [22]. Neural coupling in the delta and beta band is reported in relation to temporal prediction accuracy of the auditory beats [22–23]. Beta oscillation is also related to motor timing [24], which may provide a mechanism for time estimation of short intervals (1–2 seconds) [25]. Beta oscillation is further argued to be associated with the internal representation of time and longer produced durations (~2.5s range) are correlated with higher beta power [26]. Evidence also suggests that increased EEG/MEG delta activity occurs during anticipation and expectancy conditions [27,28].

In the present study, EEG was recorded during a mindfulness state task in order to investigate the relationship between EEG signals and over/under estimation of many-minutes-long (i.e., 15 min) intervals. Mindfulness is a manner of paying attention on purpose without judgment and in the present moment [29]. We have specifically examined mindfulness because it involves many aspects of brain function including executive function and it has recently been investigated in relation to time perception [30–32]. These studies suggest that increased attention and awareness may cause time distortion and this prediction has been confirmed by the overestimation of time in the order of short durations [30,31]. However, as outlined earlier, long-term durations have not been tested in this context. We use Fast Fourier Transform (FFT) analysis as a conventional linear approach and graph theoretical analysis as an alternative nonlinear approach for electroencephalography (EEG) analysis of the temporal judgments of relatively long intervals (i.e., 15 minutes). Graph theoretical measures can clarify some nonlinear brain function properties such as brain segregation/integration and information propagation in the brain [33]. According to the nature of graph theoretical analysis, graph measures may clarify a time perception mechanism that is not complying from a scalar framework and confirms a nonlinear mechanism such as the vector presentation of perceived time. Based on earlier EEG studies, we predicted that delta and beta activity would be related to the differences in subjective time during mindfulness. Significant differences of FFT or graph theoretical results between subjects who underestimated and overestimated time can be discussed in term of an internal clock model, as a linear approach, or vector presentation of perceived time as a nonlinear framework.

## Method

### Procedure

Two experiments were conducted, one exploratory (Study 1) and the other confirmatory (Study 2). In both experiments, participants were tested in a Faraday cage and EEG was recorded throughout the experiment. Instructions were announced via an audio recording. In the first step, participants were in the resting state in eyes open condition without substantial body and gross eye movements for 15 minutes. After 15 minutes, participants were told, using a ruler as a prop, that the elapsed time was 15 units on the ruler. In the second step, participants engaged in a mindfulness task (a body scan task, described below). After 15 minutes, participants estimated the elapsed time of the condition in comparison to the rest state and indicated their judgment of elapsed time as a unit number on the ruler. Experiment 2 was performed as a test of the reliability of the results from Experiment 1. To this end, the procedure from Experiment 1 was repeated with a smaller sample and the analyses developed in Experiment 1 were applied to the data gathered experiment 2.

### Participants' selection and ethical codes

The study was conducted in accordance with the declaration of Helsinki and had been approved by the central ethical committee of Islamic Azad University. Study was meeting criteria of the ethical committee check list; including confidentiality of participants' name during the study and in publications or sharing data, insight of participants about aim and process of study, participants' free will to leave the study in any time by any reason. In addition no financial or non-financial beneficial fair between researchers and participants was accepted. Subjects' data were recorded by subject number only, and are ready to disclose to anyone who works in the same filed or is interested. All participants were provided written informed consent after two public meeting about the aim and procedure of study and private question and answer. All of the participants had normal hearing and normal or corrected-to-normal vision, without any history of psychiatric or neurological disorders or diseases. More information about the ethical processes is accessible by contact to Professor Mehrdad Hashemi (mhashemi@iautmu.ac.ir). In regard to acceptance of ethical committee, all data (without participant’s names) have been shared as a public dataset at: https://figshare.com/articles/Over_zip/5970886. In experiment one, forty-seven healthy right-handed participants were tested and in experiment two, seventy volunteers were tested.

All of the participants had normal hearing and normal or corrected-to-normal vision, without any history of psychiatric or neurological disorders or diseases. In the first study, five participants who had noisy EEG (muscle activity and exorbitance eye blinks) were excluded and 42 participants (17 female) aged between 18 and 35 (mean: 25.79 and SD: 4.72) were included in the analysis. Overestimation of time was observed in 17 participants (6 female; aged between: 20 and 35; mean: 25.14; SD: 4.61) and underestimation was observed for 25 participants (11 female; aged between 18 and 35; mean: 26.43; SD: 4.93). In Experiment 2, participants (7 female) were between 21 and 39 years old (mean: 29.52 and SD: 6.43). Eight participants (3 female; aged between 22 and 37; mean: 29.88; SD: 5.17) estimated time as longer than 15 minutes, while the underestimation of time was observed in 9 participants (4 female; aged between 22 and 39; mean: 29.22; SD: 7.69). Table 1 shows briefly the gender and age of participants.

**Table 1:** Group information.

	Group	Number of participants	Gender	Age Range/mean/SD	t-test between groups (Age)	
					T	Sig. (2-tailed)
First study	Overestimating	17	Female: 6	20-35/25.14/4.61	-0.713	0.482
	Underestimating	25	Female: 11	18-35/26.43/4.93		
Second study	Overestimating	8	Female: 3	22-37/29.88/5.17	-0.202	0.842
	Underestimating	9	Female: 4	22-39/29.22/7.69		

### EEG acquisition and signal processing

EEG acquisition was performed in Iranian Neuro-Wave Lab. Twenty-one channels of EEG were recorded with a Brainmaster amplifier in an isolated faraday room using Ag/AgCl electrodes in the linked-ear montage. The sampling rate was 256 Hz and a 40 Hz low-pass filter was applied. EEG was recorded using a nineteen channel Electrocap® and electrodes impedance was kept under 10 kΩ. Linked-ear montage was used for recording. EEG cancelation is minimized in this montage [34] and this montage has been used in several studies of cognition [35]. Fifteen minutes of EEG was recorded in each condition. Artifact rejection was performed in two steps. First, a z-score based algorithm was applied by Neuroguide software (www.appliedneuroscience.com). This algorithm works based on amplitude and frequency. The acceptable z-scores were selected between -1.96 and +1.96 (95% accuracy). After automatic artifact rejection, the average of signal remaining was 11 min and 32 second. In the second step, remaining signals were visually inspected for artifacts. Since the stationary properties of EEG signals such as coherence and absolute power are dependent on the signal length [36], we selected 40 to 50 artifact free signal segments with length of 3 seconds. The selection was performed from the entire signals and the test-retest and split half tests for all EEG channels remained over 0.9.

Offline Fast Fourier transform (FFT) was accomplished by Neuroguide software (www.appliedneuroscience.com). Neuroguide uses an overlapping window framework for FFT analysis[37]. The number of FFT point is 256 and the overlapping ratio is 0.5. Using this approach, EEG absolute power and coherence of Delta (1–4 Hz), and Beta (12–30 Hz) was calculated. Since the beta is split into refined sub-bands in previous studies [38–40], beta band was divided into four subbands; Beta1 (12–15 Hz), Beta2 (15.5–18 Hz), Beta3 (18.5–25) and High Beta (25.5–30 Hz) and the absolute power and coherence were calculated in these subbands.

### Coherence and adjacency matrix

Although the nonlinear connectivity indices such as the phase lag has been suggested for describing the brain connectivity [41] many studies use coherence as a powerful and well-studied measure that identifies the level of coupling in cortical pathways [34,42,43]. The coherence between two time series is essentially sensitive to signal phase difference. Maximum coherence occurs when the phase difference is fixed between two signals [43]. But the coherence would be zero (or near to zero) if the phase difference between two signals was random during time [43]. Mathematically coherence is calculated by [34]:

$${\mathrm{coh}}_{\mathrm{ij}}^{2}\left(w\right)=\frac{E\left[C_{ij}{\left(w\right)}_{}^{2}\right]}{E\left[C_{ii}\left(w\right)\right]E\left[C_{jj}\left(w\right)\right]}$$,

where *C*_*ij*_ *(ω)* is the Fourier transform of the cross-correlation coefficients between EEG channels (channel i and channel j) and *C*_*ii*_ *(ω)* is co-spectrum. Coherence was calculated in 500 ms overlapping windows in the frequency domain. Then the coherences of all epochs were averaged over time.

**Fig 1:**
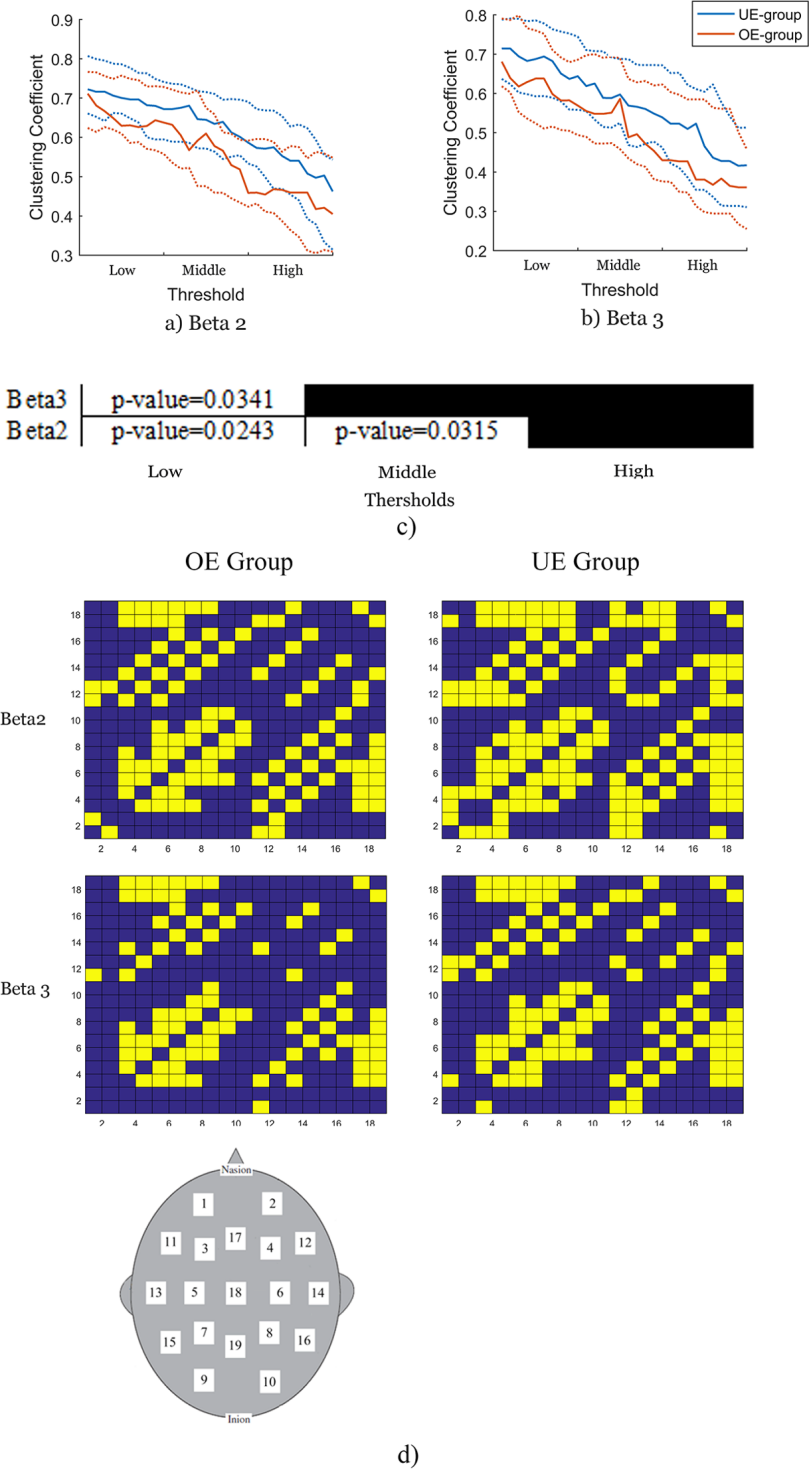
Graph features at the beta2 (15–18 Hz) and beta3 (18.5–25 Hz) sub-bands in the first study. a) Clustering coefficient at the beta2 band in terms of thresholds. UE-group exhibits higher value of clustering coefficient than OE group. Dashed lines are standard errors. b) Clustering coefficient at beta3 band in terms of thresholds. UE-group exhibits higher value of clustering coefficient than OE group. Dashed lines are standard errors. c) Clusters of significant differences between two groups at different beta sub-bands based on permutation analysis. There are significant differences at low thresholds in the beta 2 and beta 3. Significant differences are also observed at middle thresholds in beta2. d) Visualization of adjacency matrices. Each row and column demonstrates an EEG channel. The number of each channel has been shown on scalp. Yellow cubes indicate that channel *n* is connected to channel *m*. While blue cubes show unconnected nodes. Different connectivity patterns have been observed in the beta sub-bands.

An adjacency matrix is composited from connectivity measure (coherence) between all nodes (electrodes). Each row and column of an adjacency matrix is dedicated to electrodes and matrix arrays show the measure of connectivity between them (Fig 1D). Since EEG recording was accomplished by 19 distinct channels, a 19 by 19 weighed adjacency matrix was formed according to coherence between the EEG electrodes. Then, thresholds were applied and weighted graphs were changed into binary graphs. In this approach, all of the matrix arrays with higher values than threshold were replaced by 1 and others arrays were changed to zero and weighed matrix was changed to binary matrix. Previous studies indicate that since differences may have occurred in a specific threshold, a wide range of thresholds should be tested [33,41,44,45]. We investigated the thresholds in three separate clusters; low (0.2 to 0.3), mid (0.3 to 0.4) and high (0.4 to 0.5) and graph indices were investigated in these thresholds.

### Graph theoretical analysis and indices

Several topological indices have been proposed for analyzing the functional connectivity of the brain network. These measures can show the centrality and importance of nodes in graph, level of local processing and segregation, and strength of integration in graph. In this study, four well-studied measures have been considered; graph degree, clustering coefficient, transitivity and global efficiency [33,46].

In graph theory, the degree of a node shows the level of association of node in the graph. The node degree is defined as the number of edges incident to the node [47]. The average of graph degree indicates the power of connectivity in the graph and there is a direct relation between average graph degree and the number of edges in the graph [47]. Mathematically node’s degree is calculated by summation of all ones in the corresponding row of adjacency matrix.

The Clustering coefficient (C) measures the segregation and local connectivity in complex networks [33,47]. *C* is associated with the number of triangles divided by the number of all possible triangles in the matrix [33]. Evidence indicates that in neural networks, brain regions tend to make segregated circuits and share information at the local level [48]. The *C* can report the power of functional brain network for neural information processing in localized circuits [33]. Similar to *C*, transitivity is also demonstrated by the number of triangles in the matrix and can predict the level of segregation in functional brain network [33]. However, there are differences between transitivity and *C* [49]. Mathematically, transitivity is equal to 3 times of number of closed triplets in the graph divided by number of connected triples of vertices in the graph. Functionally, these two measures, transitivity and clustering, are useful indices for predicting the strength of local processing in brain network but the results typically differ.

In a binary undirected graph, the shortest path between two nodes is the path that connects them with minimum edges [47]. The average of shortest paths between all pairs of nodes is the characteristic shortest path. A graph with high ability to data transfer and integration has small characteristic shortest path and vice versa [47]. However, a problematic issue arises when we calculate the characteristic shortest path for a binary matrix. Mathematically, the shortest path between an unconnected node and other nodes is infinity and then the unconnected nodes in the graph cause ambiguity in characteristic shortest. To solve this problem, the global efficiency has been introduced. Global efficiency is sum of the inversed shortest path divided by the number of nodes. Then the unconnected nodes appear as zeros in equation. Since global efficiency is inversely related to the shortest path, a graph with a high value of global efficiency exhibits a high value of information integration [33].

All of these indices were calculated for undirected binary matrix by the open source Matlab toolbox developed by Rubinov & Sporns (2010) at http://www.brain-connectivity-toolbox.net.

### Mindfulness task

Body scan instructions were recorded and presented to participants via a loudspeaker. Participants were asked to perform the instructions. Attention is constantly directed on the body during the mindfulness task. Body scan instructions were focused on the assessment of each body region with a non-judgmental awareness. Instructions flowed and ended with attention on the body as a whole. Participants were asked to avoid movements. The mindfulness task was performed at eye open condition and participants were asked to keep their eyes open during task. The total time of body scan task was 15 minutes. Since imagination of movement suppresses the beta waves in motor cortex [50], the mindfulness task may change the amplitude of beta and this suppression can be associated with task maintenance.

### Statistical and clustering analysis

Absolute powers, coherences and graph indices in delta (1–4 Hz) and four divided beta subbands (i.e., beta1 (12.5–15), beta2 (15.5–18), beta3 (18.5–25) and high beta (25.5–30)) were compared using nonparametric permutation test [51] between the underestimation and overestimation groups in two conditions; rest and mindfulness. Absolute power was compared on the selected relevant electrodes i.e. C3, C4, O1, O2, Fz, Cz, and Pz. 5000 random shuffles were done in each permutation test and independent permutation tests were separately performed for absolute powers and graph indices. Absolute powers in five bands (delta and four beta bands) and two conditions (rest and mindfulness) were compared between groups. False Discovery Rate (FDR) [52] has been used to correct for multiple comparisons.

Cluster-based nonparametric permutation test [51] was performed for comparing the graph indices in various thresholds. We compared the graph indices in three separate threshold clusters; low, mid and high. In each cluster ten thresholds were compared and cluster-based permutation test was accomplished. The significance level for the p-values was selected under 0.05. All of the permutation tests were performed using MATLAB 2016b. K-means approach was used for clustering the data into two groups. We used clustering approach to find the effectiveness of electrophysiological measures in the separation of groups. The k-means results may indicate which measures (linear or nonlinear) can make the difference between the UE and OE groups for an untrained machine. Two dimension k-means was performed and all possible pairs of the significant differed measures were used as inputs. Then the efficiencies of clustering with various inputs were compared and best accuracy was demonstrated. Clustering efficiency was calculated by:

$$E=\frac{\left|P_{1u}-P_{1O}+\left|P_{2u}-P_{2O}\right|\right|}{100}$$

Where *P*_*1u*_ is the probability of underestimating with label 1 and *P*_*1O*_ is probability of overestimating with label 1 and so on. K-means analysis was accomplished by MATLAB 2016a.

## Results

### Behavioral results

In Experiment 1, 17 of 42 participants estimated the duration of mindfulness state as longer (Mean: 19.82, SD = 3.64) than 15 minutes (OE group) and 25 participants as shorter (Mean = 11.24, SD: 2.09) than 15 minutes (UE group). In Experiment 2, 9 participants estimated time shorter than 15 minutes, while 8 participants overestimated the time. There was no significant difference in age between the two groups either in the first (t = -0.713 and p- = 0.482) or second (t = 0.202, p- = 0.842) experiment.

### FFT results

**Fig 2:**
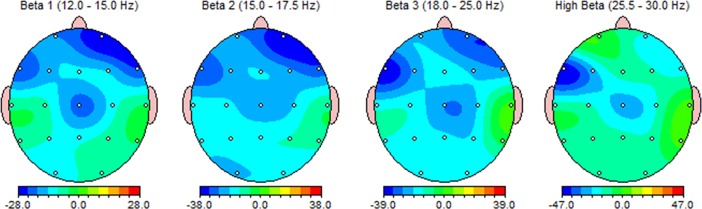
Percent differences of absolute power between two groups, in the first study. Average absolute power of OE-group was lower than the absolute power of UE-group.

Nonparametric permutation test and *FDR* correction indicated that there were no significant differences between two groups during rest state (control) condition. In the mindfulness condition, there were significant differences in absolute power between the two groups (OE-group vs. UE-group) at beta3 on Cz (t = 3.13, p = 0.003), Pz (t = 2.98, p = 0.0001), O1 (t = 3.25, p = 0.0001), O2 (t = 2.64, p = 0.004), C3 (t = 2.36, p = 0.02), C4 (t = 3.12, p = 0.003)and at high beta on Cz (t = 3.13, p = 0.0001) and C4 (t = 2.64, p = 0.003). For all of these electrodes, OE-group exhibited a lower value of absolute power than UE-group (Fig 2). This result was partially confirmed in the second (confirmatory) experiment. In the confirmatory experiment, comparisons were performed only on electrodes with significant differences detected in the exploratory experiment (Experiment 1). In the beta3 band, OE group exhibited significantly lower value of the absolute power than UE group at Cz, C4 as observed in Experiment 1. But there were no significant differences at the other electrodes. OE group also exhibited significantly lower absolute power of the high beta at Cz electrode. This result was similar to the result gathered in Experiment 1.

**Fig 3:**
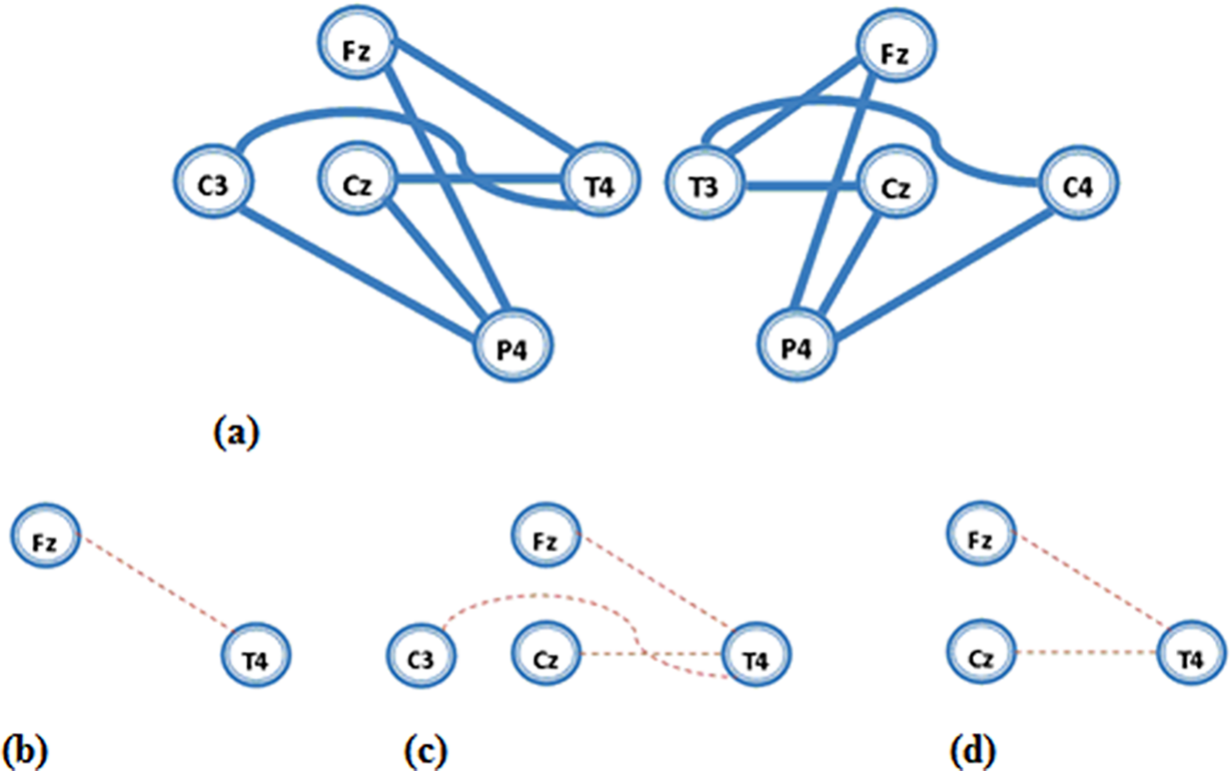
a) Two networks that were investigated in coherence analysis. At the left network, six pairs of electrodes (Fz-T4, Fz-P4, Cz-T4, Cz-P4, C3-T4 and C3-P4) were evaluated. At the right network six electrode pairs (Fz-T3, Fz-P4, Cz-T3, Cz-P4, C4-T3 and C4-P4) were evaluated. Dashed line indicates significant differences of coherence between two groups (after Bonferroni correction). Results showed that the OE group exhibited significantly lower value of coherence than the UE group at centro-frontal electrodes and right temporal electrode (T4). These differences occurred at b) beta2, c) beta3 and d) High beta.

In the exploratory experiment, significant differences in coherence also in the selected pathways (Fig 3A) were observed between OE and UE groups. Permutation test indicated that this difference was restricted to the beta2, beta3 and high beta bands on the tempo-central and tempo-frontal electrodes (Fig 3B, 3C and 3D). In these regions, the OE group exhibited significantly lower coherence than the UE group. However, the results could not be replicated in the confirmatory experiment.

### Graph and brain network analysis results

In the exploratory experiment, permutation test indicated that the OE and the UE groups exhibited significant differences in clustering coefficient in the beta sub-bands. In the beta2 and beta3 sub-bands, the UE group exhibited a higher clustering coefficient than the OE group (Fig 1). No significant difference was observed between the two groups in the beta1, high beta and delta bands. In the other parameters i.e. global efficiency, degree and transitivity, UE group exhibited higher values than OE group, but these were not significant. In the confirmatory experiment, striking differences of clustering coefficient were observed at the beta3 band respective to low thresholds.

### Clustering results

**Fig 4:**
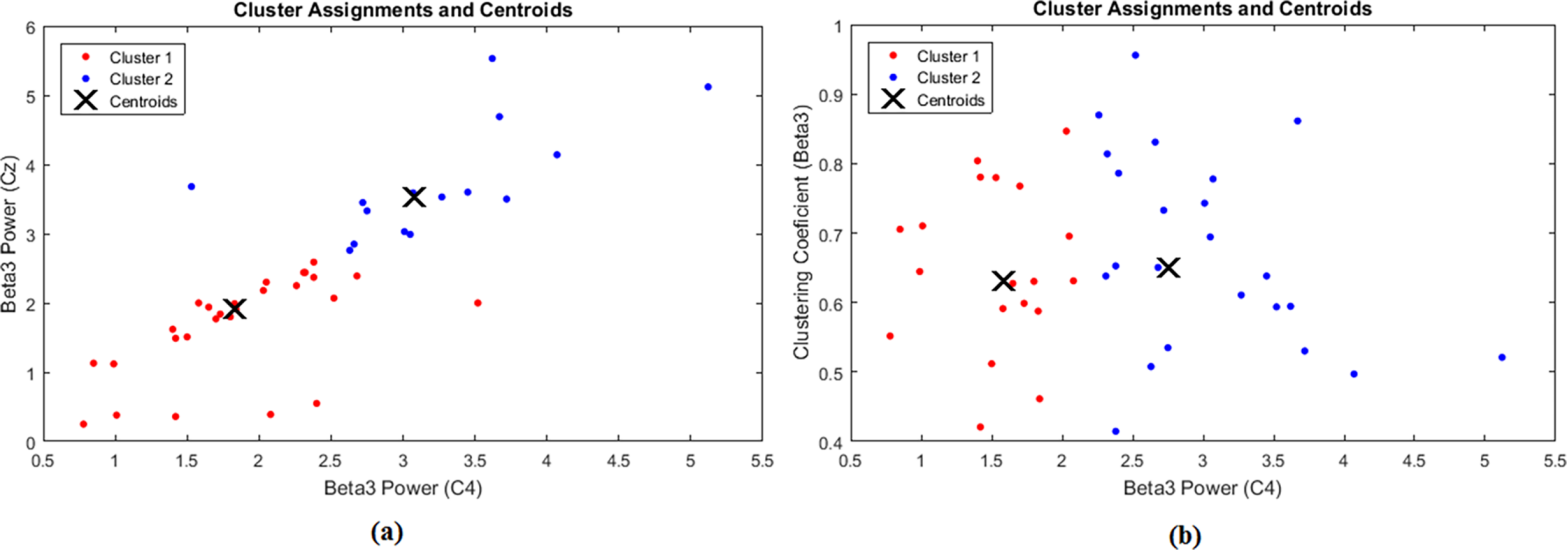
K-means clustering results of the first experiment. a) Clustering by absolute powers of C4 and Cz at beta3. b) Clustering by absolute power of C4 and clustering coefficient at beta3 bands. Accuracy of clustering is improved using both absolute power and graph index (clustering coefficient) as inputs.

Best clustering results were achieved at beta3 band (Fig 4). The efficiency of clustering, using k-means was more reliable when both results of graph theory approach (clustering coefficient) and FFT (absolute power on C4) were used as inputs (efficiency = 0.82) (Fig 4B). In this condition, the accuracy of prediction was 77% for the OE group and 64% for the UE group. It means that when we used these inputs, the participants who were assigned with index 1 belonged to OE group with possibility of 77% and were members of the UE group with possibility of 23%. This accuracy was lower for index 2. Participants who were assigned by index 2 belonged to the UE group with possibility of 64% and appertained to the OE group with possibility of 36%. Clustering accuracy and efficiency was reduced for other pairs of inputs in beta3 and high beta. For example, when absolute powers of Cz and C4 were used as inputs, the efficiency of clustering reduced to 0.64 (Fig 4A).

## Discussion

The current study compared the EEG powers and functional connectivity of EEG network between two groups with different judgments about fixed multi-minutes (i. e. 15 min) duration. The results indicate that participants who perceived time as shorter than the physical time, exhibit higher powers of beta and also higher coherence particularly at central regions. Furthermore, the underestimating group shows higher clustering coefficient in the beta sub-bands. We discuss our results in terms of two main aspects. First, FFT analysis as a linear approach, may clarify the relation between localized brain activity (i.e., basal ganglia (BG)) and time estimation. Second, non-localized properties of psychological interval timing (as emphasized in the intrinsic models) have been addressed and discussed via graph analysis results. At the end, we discuss the scalar or vector properties of subjective time.

### Beta activity, BG-thalamocortical circuits and time estimation

FFT results showed that the OE group and the UE group have exhibited different beta and high beta absolute power during the mindfulness state. Beta activity at central areas is commonly related to sensory-motor rhythms [53]. Functionally, the activity of sensory-motor beta is negatively associated with activity of the BG-thalamocortical circuits [53,54]. Therefore, abnormalities in the beta power are frequently reported in disorders associated with impairment of the BG-thalamocortical circuits; such as Parkinson’s disease [55], attention deficit and hyperactivity disorder [56] and also stuttering [57,58]. BG-thalamocortical circuits also play a critical role in the scalar timing models as an internal clock-accumulator structure. According to internal clock model, increased activity of striatum causes an increase of clock speed and leads to overestimation of time [6,7]. Therefore, as an example, underestimation of short durations has been reported among persons with Parkinson’s disease [59]. Another study suggests that they exhibit increased beta activity in centro-frontal regions [60] and these symptoms are also reported in relation to decreased activity of striatum [61]. Moreover, the direct relationship between beta desynchronization and activity of cortico-basal motor structures has been observed [62].

In this research line, recent findings indicate that there is a significant relationship between beta power, activity of striatum and estimation of time at several hundreds to multi thousands of milliseconds [24,25,27,63]. In agreement with previous reports, our results show that UE group has enhanced beta power compared to the OE group. Since the significant differences between two groups have been observed during the mindfulness task but not during the rest state, the differences of beta amplitude between the OE and the UE groups may be considered in relation to motor imagery and preparation [51] involved in the mindfulness task. Then, we can assume that mindfulness level or execution of instructions modulates beta activity and also time perception. It is consistent with Kononowicz & van Rijn study that suggests the motor preparation and EEG beta synchronization/desynchronization are associated with time production [27]. According to internal clock model and regarding to inverse relation between beta power and activity of BG-thalamocortical circuits [62], we can argue that the UE group, on average, exhibits lower activity of BG-thalamocortical circuits than the OE group. Therefore, the subjective time is contracted. This argument is in agreement with the scalar timing model and suggests the applicability of this model to relatively longer durations (i.e. 15 min).

### Non-localized properties of time perception and graph theory approach

Graph theoretical analysis is a nonlinear approach for investigating a highly complicated system such as the brain [33]. Graph indices can clarify some nonlinear behavior of the systems that cannot be accounted by simple linear methods [47]. Clustering coefficient is directly correlated with brain segregation [33,46]and there is no linear relation between global brain activity and level of clustering coefficient. Instead, clustering coefficient may be altered by other topological properties of complex network (e. g. social network) such as information propagation and local coupling [64]. Since there is a major debate about linear [65] or nonlinear [13] nature of perceived time, a graph theoretical approach was used for investigating possible nonlinear differences of brain activity between the OE and UE groups.

Since there were significant differences in clustering coefficient between two groups, we suggest that nonlinear properties of brain function such as information sharing and brain segregation have been involved in perception of longer durations (i. e. 15 min). Although the clustering coefficient may predict the local connectivity in the brain, there is no linear correlation between clustering coefficient and activity of brain. Mathematically, the clustering coefficient is associated with number of triangles in the network [47]. When a node shares data with two nodes and those nodes are connected together, then a cluster has been made. The ratio of connected neighbors to all of the neighbors is equal to clustering coefficient. Therefore, a node can share information with many nodes and clustering remains very low. On the other hand, a node may share information only with two nodes and maximum clustering coefficient can be achieved (i.e., 1). Then, the nonlinearity of complex brain network segregation cannot be directly derived from local brain activity and specific neural pathways are required for augmenting the clustering coefficient.

We tried to examine a classification approach in order to separate OE than UE group using linear (FFT) and nonlinear (graph theory) measures. K-means approach was used for unsupervised clustering of participants. Results indicate that beta3 absolute powers (at C4, O1) can be used as predictors for the UE group labeling. However, in the case of the OE group, there was no an acceptable prediction. Efficiency of prediction is improved using both beta3 clustering coefficient and beta3 power at C4 as inputs. Increased prediction accuracy using clustering coefficient can suggest that time estimation at a long term duration may also correlate with the segregation at cortico-cortical pathways and non-localized functions. Consistent with this argument, abnormal beta segregation has been observed in disorders related to abnormal timing such as stuttering [58] and Parkinson’s disease [66]. This result is in agreement with the model which suggests that there is no linear metric of time and time may be perceived via patterns presented at the network level [13].

### Scalar or vector subjective timing

**Fig 5:**
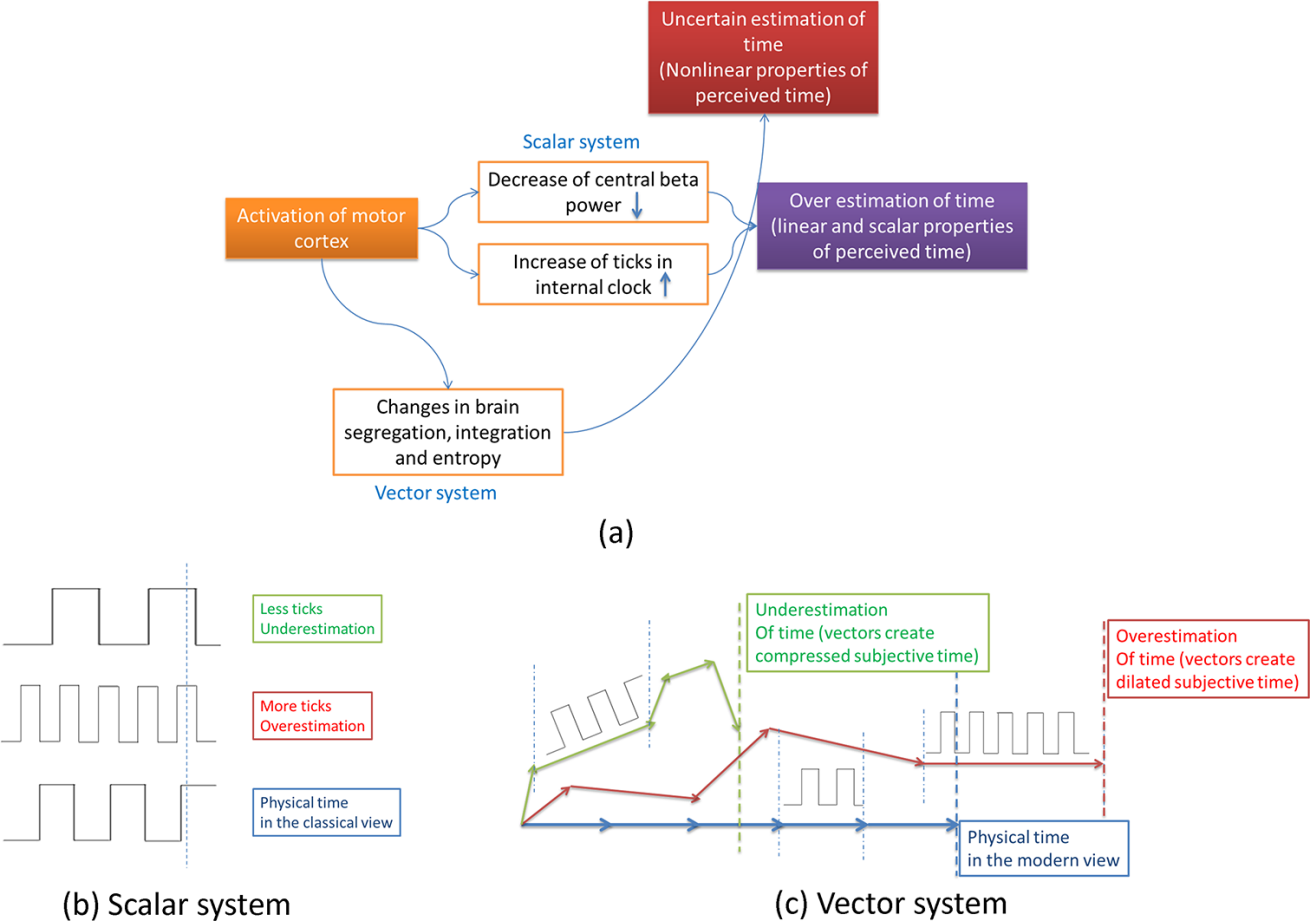
a) The scalar subjective timing vs. the vector subjective timing. In the scalar system, higher activity of BG-thalamo-cortical pathways and lower power of motor related beta lead to overestimation of time. However, regarding the vector system, the estimation of time may involve non-linear brain properties such as brain integration, segregation and information processing (entropy). b) In the scalar system, numbers of ticks are linearly related to subjective time. More ticks cause to overestimation of time while underestimation of time is accompanied with less ticks occurrence. c) According to vector system, subjective time in each moment has two main aspects: length and direction. Length of each vector may be demonstrated by the number of ticks (generated in BG-thalamo-cortical pathways). However, direction of vectors may be indicated by nonlinear parameters such as brain integration/segregation and information processing. When direction of moment vectors is parallel to direction of physical time, overestimation of time is occurred. While, long length vectors of subjective time may perceive very short when there is a large angle between physical and subjective time.

According to results, although activity of BG-thalamocortical pathway (as a dedicated module) is important in relation to time perception mechanisms, properties of functional activity of brain network; such as segregation of beta network are significant in perception of time. Functional EEG segregation is closely related to efficiency and speed of information processing in the cortical brain network [33]. Therefore, vector properties (i.e., information and speed) may also have a role in human perception of long time duration. Fig 5 briefly indicates that time may be perceived via scalar or vector systems. Although scalar system justifies overestimation of time related to hypo-activity of motor beta, other non-predictable features of time perception may be explained by the vector system. According to the vector subjective timing system, information processing in the brain network (brain integration, segregation and entropy) may be altered by activation/deactivation of time module related regions of the brain. *We can assume that perceived durations consist vectors of time in each moment (instead of scalar ticks)*. Each vector has a direction and a length. Activity of internal clock (related to linear properties of time) may change the length of vectors but direction of vectors may be determined by information processing in the brain. In this framework, long length vectors of subjective time may be perceived very short when there is a large angle between physical and subjective time (Fig 5C).
